# Loss of OXGR1 function uncouples intrarenal RAS activation from pendrin and αENaC regulation and blunts angiotensin II-induced hypertension

**DOI:** 10.3389/fphar.2026.1869136

**Published:** 2026-08-31

**Authors:** Pilar Cárdenas, Juan Castillo-Geraldo, Lilian Caroline Gonçalves de Oliveira, Nicolas Muñoz, Rodrigo Yokota, Marina de Moura Bello, Gabriel Joo-Mulsow, Luis Michea, Dulce Elena Casarini, Alexis A. Gonzalez

**Affiliations:** 1 Laboratory of Biological Chemistry, Institute of Chemistry, Pontificia Universidad Católica de Valparaíso, Valparaíso, Chile; 2 Nephrology Division, Department of Medicine, Universidade Federal de São Paulo (UNIFESP/EPM), Sao Paulo, Brazil; 3 Facultad de Medicina, Instituto de Ciencias Biomédicas, Universidad de Chile, Santiago, Chile; 4 Centro de Investigación Interdisciplinaria en Biomedicina, Biotecnología y Bienestar (CID3B), Pontificia Universidad Católica de Valparaíso, Valparaíso, Chile

**Keywords:** angiotensin, hypertension, montelukast, oxoglutarate receptor knockout, sodium transport

## Abstract

**Background:**

Activation of the intrarenal renin–angiotensin system (RAS) is a key mechanism driving sodium retention and hypertension. In the distal nephron, locally generated angiotensin II (Ang II) promotes Na^+^ reabsorption through coordinated regulation of the epithelial Na^+^ channel (ENaC) in principal cells and electroneutral NaCl transport mediated by pendrin and the Na^+^-dependent Cl^−^/HCO3^−^ exchanger (NDCBE) in intercalated cells. Emerging evidence identifies α-ketoglutarate as an intrarenal paracrine signal acting through oxoglutarate receptor 1 (OXGR1), which is highly expressed in collecting duct intercalated cells. We hypothesized that OXGR1 is required to couple intrarenal RAS activation with collecting duct Na^+^ transport and blood pressure regulation during Ang II-dependent hypertension.

**Methods:**

mice were subjected to chronic Ang II infusion (400 ng·min^-1^·kg^-1^, 14 days) using two approaches: pharmacological OXGR1 blockade with montelukast (3.5 μg ·min^-1^·kg^-1^) and global OXGR1 genetic deletion (OXGR1^−/−^). Blood pressure, Na^+^ balance, and the renal abundance of pendrin, NDCBE and αENaC were assessed. Intrarenal RAS activity was evaluated by measuring Ang I (renin activity), Ang II, ACE activity and (pro)renin receptor (PRR).

**Results:**

Ang II infusion increased blood pressure, promoted antinatriuresis, and elevated intrarenal Ang II, ACE activity, PRR, pendrin, and αENaC protein abundance. In contrast, both montelukast-treated wild type and OXGR1^−/−^ mice infused with Ang II displayed attenuated hypertensive responses, reduced Na^+^ retention, and failed to increase intrarenal Ang II, pendrin, or αENaC abundance.

**Conclusion:**

These findings identify OXGR1 as a critical regulator linking intrarenal RAS activation to distal nephron Na^+^ transport and suggest that OXGR1-dependent signaling contributes to sodium retention and the development of Ang II-induced hypertension.

## 1 Introduction

The kidney plays a dominant role in the long-term regulation of arterial pressure through its capacity to modulate Na^+^ excretion and extracellular fluid volume (Adamczak et al., 2002). Among the endocrine and paracrine systems regulating renal Na^+^ balance, the renin-angiotensin system (RAS) occupies a central position (Fountain and Lappin, 2018). In addition to the circulating RAS, all components required for Ang II generation, including angiotensinogen, renin, and angiotensin-converting enzyme (ACE), are expressed within the kidney (Kobori et al., 2003; Kobori et al., 2004). In addition to Ang II accumulation, renal Ang II is further augmented by intrarenal angiotensinogen production, which correlates with intrarenal RAS activity and blood pressure in both experimental models and humans (Kobori et al., 2003; Kobori et al., 2004). Thus, this intrarenal RAS can sustain intrarenal Ang II signaling independently of systemic levels and act as an active, self-reinforcing network (Nehme et al., 2019). Moreover, chronic Ang II infusion experimental models have demonstrated that intrarenal Ang II generation is not only amplified but also required for the development of hypertension (Giani et al., 2015; Gonzalez-Villalobos et al., 2013). This amplification is driven by enhanced local ACE activity, increased angiotensinogen expression, and sustained AT1 receptor signaling within the nephron (Gonzalez-Villalobos et al., 2008a; Gonzalez-Villalobos et al., 2008b; Prieto et al., 2021). Also, Ang II by itself can upregulate prorenin and PRR, which work as a receptor for renin and prorenin, increasing the renin catalytic activity to generate Ang I from angiotensinogen (Nguyen and Burckle, 2004).

Intrarenal RAS activity is spatially heterogeneous. The collecting duct (Gonzalez et al., 2017; Peng et al., 2017; Pri et al., 2011; Gonzalez et al., 2011), expresses renin, prorenin, and the (pro)renin receptor (PRR) (Nguyen and Burckle, 2004). ACE and AT1R are also present in this segment, enabling local Ang II generation and autocrine/paracrine signaling (Kobori et al., 2004; Song et al., 2016; Prieto et al., 2017; C and offman, 2010). In the collecting duct, Ang II directly activates the epithelial Na^+^ channel (ENaC) of principal cells, enhancing Na^+^ reabsorption (Zaika et al., 2013). In addition, the intercalated cells, traditionally considered specialized regulators of acid-base balance, play a central role in NaCl handling. These cells express pendrin (SLC26A4), an apical Cl^−^/HCO_3_^−^ exchanger that operates in concert with the Na^+^-dependent Cl^−^/HCO_3_^−^ exchanger NDCBE to mediate electroneutral NaCl reabsorption, functionally coupled to ENaC-driven electrochemical gradients (Wall et al., 2020). Pendrin expression is modulated by dietary NaCl. Ang II upregulates pendrin expression in intercalated cells (Gonzalez-Villalobos et al., 2013) and volume depletion, mineralocorticoid excess, and Ang II stimulation are important up regulators of pendrin *in vivo* (Verlander et al., 2011; Verlander et al., 2006).

α-ketoglutarate has emerged as a key intrarenal paracrine metabolic signal that modulates collecting duct function. α-ketoglutarate is generated through the tricarboxylic acid cycle and renal gluconeogenesis, and is freely filtered and secreted by proximal tubule cells into the tubular lumen (Vishwakarma, 1963; Guder, 1976; Guder, 1979). α-ketoglutarate accumulates in distal nephron segments, where it exerts biological effects independent of its intracellular metabolic functions (Tokonami et al., 2013), via its receptor OXGR1 (also known as GPR99) (Wittenberger et al., 2002). OXGR1 is highly expressed in collecting duct intercalated cells, where it senses luminal α-ketoglutarate and regulates bicarbonate secretion and chloride absorption. In addition, the α-ketoglutarate-OXGR1 signaling has been implicated in Na^+^ handling. Lazo-Fernandez and colleagues demonstrated that α-ketoglutarate stimulates pendrin-dependent chloride absorption in the cortical collecting duct via protein kinase C-dependent mechanisms (Lazo-Fernandez et al., 2015; Lazo-Fernandez et al., 2018). Our group demonstrated that α-ketoglutarate upregulates PRR expression in collecting duct cells via OXGR1 activation, leading to increased intratubular Ang II formation and enhanced Na^+^ reabsorption under high-glucose conditions (Guerrero et al., 2021). These findings provided the first mechanistic link between metabolic signaling, intrarenal RAS amplification, and distal nephron Na^+^ retention. More recently, we showed that OXGR1-dependent PRR upregulation in collecting ducts contributes to Na^+^ balance in kidneys with reduced blood flow (Cardenas et al., 2024). Given the central role of Ang II in driving distal Na^+^ reabsorption through αENaC and pendrin, and the emerging role of OXGR1 in regulating intratubular Ang II formation, these results raised the possibility that OXGR1 serves as a metabolic amplifier of intrarenal RAS signaling in Ang II-dependent hypertension.

Previous evidence from our laboratory suggests that OXGR1 may function as an important amplifier of the intrarenal RAS by increasing PRR expression, thereby enhancing renin activity within the collecting duct and promoting intratubular Ang II formation. Based on these observations, we hypothesized that OXGR1 signaling is required for Ang II-induced sodium retention, blood pressure elevation, and activation of collecting duct NaCl transport through coordinated regulation of αENaC and the pendrin/NDCBE transport system. To test this hypothesis, we first evaluated whether pharmacological inhibition of OXGR1 with montelukast (Kanaoka et al., 2013; Guerrero et al., 2021) attenuates hypertension, sodium retention, and intrarenal RAS activation in mice infused with a pressor dose of Ang II infusion for 14 days. Intrarenal RAS activity was assessed by measuring renal Ang I and Ang II levels, ACE activity, PRR protein abundance, and the expression of αENaC, pendrin, and NDCBE. To further determine whether these effects were specifically mediated by OXGR1, parallel studies were performed in global OXGR1 knockout mice subjected to chronic Ang II infusion under the same experimental conditions. Our findings demonstrate that OXGR1 is required for the full hypertensive response to Ang II and for the associated increases in pendrin and αENaC in the collecting duct.

## 2 Materials and methods

### 2.1 Animals and experimental design

Experimental Animals. All protocols were performed in accordance with the Institutional Animal Care and the Bioethical Committee of the Pontificia Universidad Católica de Valparaíso (BIOEPUCV-BA 895-2025), under international guidelines and regulations for animal use. Twelve-week-old male C57BL/6 mice were placed under the following conditions: light-dark cycle (12 h), temperature of 21 °C, humidity of 50%, adequate ventilation, noise free, and food and water *ad libitum*. Mice were divided according to the specific protocol using n = 6 animals per group. The study design included two protocols: 1) a pharmacological modulation of OXGR1 signaling using Montelukast, administered either alone or in combination with angiotensin II (Ang II) infusion over a 14-day period; and 2) OXGR1^−/−^ mice with angiotensin II (Ang II) infusion over a 14-day period. OXGR1^−/−^ mice with global deletion of the OXGR1 protein were a gift from Dr. Joseph E. Kerschner (Medical College of Wisconsin). The colony of OXGR1^−/−^ mice (C57BL/6J background) was established from breeding pairs of OXGR1 heterozygous (OXGR1^+/−^) mice. Heterozygous mice and wild-type (WT) mice showed no difference in previous tests of blood pressure, water and food intake, and urinary flow, among other physiological parameters; thus, sham-operated OXGR1^+/−^ and sham WT-operated mice were also used as controls and called “sham” for the rest of the study. Genotype was verified by PCR reaction of tail genomic DNA. Primer pairs UTT069-21 (5′-GAG​CCA​TGA​TTG​AGC​CAC​TG-3′) and UTT069-25 (5′-CAC​CAC​TGG​CAT​AGT​AAT​GG-3′) generated a 294 bp OXGR1 specific fragment which is present in wild type but is absent in mutant allele, and UTT069-3 (5′-CAG​AGC​CAT​GCC​TAC​GAG-3′) and GT (5′CCC​TAG​GAA​TGC​TCG​TCA​AGA-3′) amplified a 378 bp fragment specific to the selection cassette in the mutant allele.

### 2.2 Chronic infusion protocols

Continuous delivery of Ang II and/or Montelukast was achieved using subcutaneously implanted osmotic minipumps (ALZET model 2002). Animals were anesthetized with 2.5% isoflurane prior to implantation. Pumps were inserted through a small dorsal incision on day 0. Ang II was administered at a rate of 400 ng·min^−1^ kg^−1^. Montelukast was delivered at 3.5 μg·min^−1^ kg^−1^, which is equivalent to approximately 5 mg/kg/day when expressed as a daily dose. This dose is consistent with previously published pharmacological studies using montelukast in rodent models of renal disease (Khodir et al., 2014; Khodir et al., 2016). Both compounds were dissolved in phosphate-buffered saline and infused at a constant rate of 0.5 μL/h for 14 days.

### 2.3 Blood pressure measurements

Systolic and diastolic arterial blood pressure were measured in conscious mice using a non-invasive tail-cuff system (CODA, Kent Scientific, Torrington, CT, USA), which simultaneously records systolic, diastolic, and mean arterial pressure as well as heart rate. Mice were acclimated to the procedure for 5 consecutive days before baseline measurements. For each session, animals were briefly anesthetized with isoflurane to facilitate placement into a temperature-controlled chamber maintained at 36 °C. After complete recovery, blood pressure recordings were obtained in conscious mice. Ten consecutive measurements were collected per animal, and the average value was used for analysis. Blood pressure was assessed on days 3, 5, 7, and 13 of the experimental protocol (Gonzalez et al., 2011).

### 2.4 Metabolic cage studies

To evaluate renal excretory function, animals were individually housed in metabolic cages for 24-h periods before treatment initiation and at day 13. Parameters recorded included urine output, water consumption, and food intake. During these periods, animals were monitored at regular intervals (every 3–6 h) to ensure physiological stability. Urinary sodium and chloride concentrations were measured in duplicate, using the Chloride liquicolor TPTZ method (Humana, Germany), according to manufacturer’s instructions for the semimicro protocol, adjusted for diluting 10 μL of urine in deionized distilled water.

### 2.5 Saline challenge test

The natriuretic responses to extracellular fluid volume expansion were determined during the morning of day 13 to evaluate the effect of Ang II infusion with or without Montelukast or in OXGR1^−/−^mice. At 9:00 a.m. mice were lightly anesthetized with isoflurane, injected i.p. with a volume of 37 °C 0.9% NaCl equivalent to 10% of their body weight (v/w), and placed immediately in metabolic cages. Urine was collected at 2, 4 and 6 h. Since no significant differences were detected among the experimental groups at intervals od 2 and 4 h, the cumulative 6-h sodium balance - calculated as the amount of sodium administered minus the amount excreted - provided a more sensitive and physiologically relevant assessment of renal sodium retention. Na^+^ concentration was measured in duplicate, using the Chloride liquicolor TPTZ method (Humana, Germany), according to manufacturer’s instructions for the semimicro protocol, adjusted for diluting 10 μL of urine diluted in deionized distilled water.

### 2.6 Protein expression analysis

Renal protein abundance was evaluated by immunoblotting. Tissues were homogenized (40 μg protein) and electrophoretically separated on a precast NuPAGE 10% Bis-Tris gel (Novex) at 200 v for 45 min, followed by semi-dry transference to a nitrocellulose membrane (Invitrogen) using iBlot (Invitrogen, Carlsbad, CA, USA). Blots were blocked with non-fat milk at RT for 3 h, incubated overnight with specific primary antibody at 4 °C, incubated with the corresponding secondary antibodies (1:5,000 dilutions) at room temperature for 45 min, and then analyzed by normalization against β-actin, used as a housekeeping gene. PRR protein levels were detected using a polyclonal rabbit anti Pendrin (G-11; sc-5181108, Santa Cruz Biotechnology) at 1:200 dilutions; anti NDCBE (LX-2; sc-100672; Santa Cruz Biotechnology) at 1:100 dilutions; anti PRR (ATP6AP2, 1:200; Cat. #HPA003156, Sigma-Aldrich, St. Louis, MO, USA) at 1:200 dilutions, allowing recognize full length and clavated forms of PRR; αENaC (Ls-c150488, LS Bio) at 1:500 dilutions; anion exchanger 4 (AE-4 CSB-CSB-PA842743LA01HU, Cusabio, TX, USA) at 1:500 m dilutions and b-actin (sc-47778, Santa Cruz Biotechnology) at 1:200 dilutions. All blots presented in figures were cropped and original figures are available by request and presented to the reviewers to check identity and molecular weight properly.

### 2.7 Immunohistochemical analysis

Kidney tissues were harvested, fixed in Bouin (Saturated Aqueous Picric Acid), embedded in paraffin, sectioned, and stained with specific antibodies Kidney sections (3 μm) were stained with anti-pendrin at 1:10 dilutions (G-11, Cat. # sc-518108, Santa Cruz Biotechnology, Dallas, TX, USA) and anti-αENaC Ls-c150488, LS Bio) at 1:10 dilutions. The reaction was visualized using the ImmPACT DAB Substrate Kit (Vector Laboratories, Cat. # SK-4105, Newark, CA, USA). The images were obtained using a Nikon Eclipse-50i microscope (Nikon Instruments Inc., Melville, NY, USA) and were digitized using the NIS-Elements BR version 4.0 from Nikon. Immunostaining intensity was quantified as the ratio of DAB-positive (brown) pixels to total pixels across 4 representative fields per mouse including outer and inner medulla.

### 2.8 Determination of renal angiotensin peptides

Renal angiotensin peptides were performed as described previously (Abreu et al., 2022). On the day of extraction, a frozen kidney fragment (50 mg/0.5 mL of buffer) was homogenized for 30–45 s, in a cooled 2 mL microcentrifuge tube, with stainless steel beads in it. On the day of extraction, a frozen kidney fragment was homogenized for 30–45 s in a cooled 2 mL microcentrifuge tube using a Potter-Elvehjem homogenizer. For this, ice-cold modified RIPA lysis buffer (50 mM Tris-HCl pH 8.0, 50 mM NaCl, 1% v/v NP-40, 0.5% w/v sodium deoxycholate, and 0.1% w/v SDS), supplemented with the following protease inhibitors: leupeptin (5 μg/mL), pepstatin A (5 μg/mL), PMSF (1 mM), benzamidine (0.1 mg/mL), and aprotinin (10 μg/mL), was used for homogenate preparation. Samples were centrifuged at 15,000 × g for 15 min at 4 °C, and the recovered supernatant, considered as the total lysate, was used for angiotensin quantification. The samples were prepared for solid-phase extraction with the addition of the Sar1-Leu8 Ang II internal standard and thus extracted on Sep-PakC18 3 cc columns (Waters, USA). Extraction control was done using a tenfold concentration with the peptides of interest (Ang I, Ang II, Ang-(1–7) and Sar-Leu Ang II) (Sigma, USA). The peptides were separated on a Poroshell 120 EC-C18 reverse-phase column (3.0 × 50 mm, 2.7 μm, Agilent Technologies, USA) using a linear gradient of mobile phase A (H2O, trifluoroacetic acid 0.1%) and mobile phase B (methanol, trifluoroacetic acid 0.08%) in Exion LC AE HPLC (Sciex, USA), and subsequently injected into the Mass Spectrometer coupled to HPLC, AB Sciex 6500 QTRAP (Sciex, USA). The detection of the peptides was performed with the multiple reaction monitoring (MRM) according to their mass-to-charge ratio (m/z) and retention, as previously described. Calculations were performed from a calibration curve (with matrix) in the range of 1 fmol–100 fmol per injection. Quantification of the samples was determined by calculating the peak area displayed for the mass of the target ions in the spectrometer, referenced to the known concentration of standard peptides acquired under the same conditions over the same time. The results were analyzed by Sciex OS 3.4.5.828.

### 2.9 Angiotensin-converting enzyme activity

Freeze-dried samples were resuspended in 1 mL of 100 mM sodium borohydride buffer, pH 7.2, containing 340 mM sucrose and 300 mM NaCl, and a protease inhibitor cocktail (complete mini EDTA-free; Roche - Ref: 11836170001 - Lot: 46548400) was added. The protein concentration was determined. ACE activity was determined by a fluorometric method using Z-Phe-His-Leu-OH as substrate. Briefly, 10 µL of the homogenate sample was incubated with the substrate diluted in borate buffer containing ZnSO_4_ at 37 °C for 15 h. Enzymatic activity was interrupted by the addition of 0.28 M NaOH. The dipeptide His-Leu released by cleavage reacted with orthophthaldialdehyde, forming a fluorescent adduct. After 10 min, the reaction was terminated by the additionof 3 M HCl Fluorescence intensity was measured using a spectrofluorometer (Infinite® 200 Pro, Tecan) with excitation at 360 nm and emission at 465 nm. ACE activity was normalized to total protein concentration and expressed as nmol/min/mg of protein.

### 2.10 Statistical analysis

Data are presented as mean ± SEM. Distribution normality was evaluated using the Shapiro–Wilk test. Comparisons among multiple groups were performed using one-way ANOVA, followed by appropriate *post hoc* tests. Pairwise comparisons were conducted using unpaired t-tests where applicable. Statistical significance was defined as p < 0.05.

## 3 Results

### 3.1 Protocol 1: effect of montelukast on Ang II-infused hypertensive mice

#### 3.1.1 Montelukast blunted the increases in blood pressure induced by chronic Ang II infusion

**FIGURE 1:**
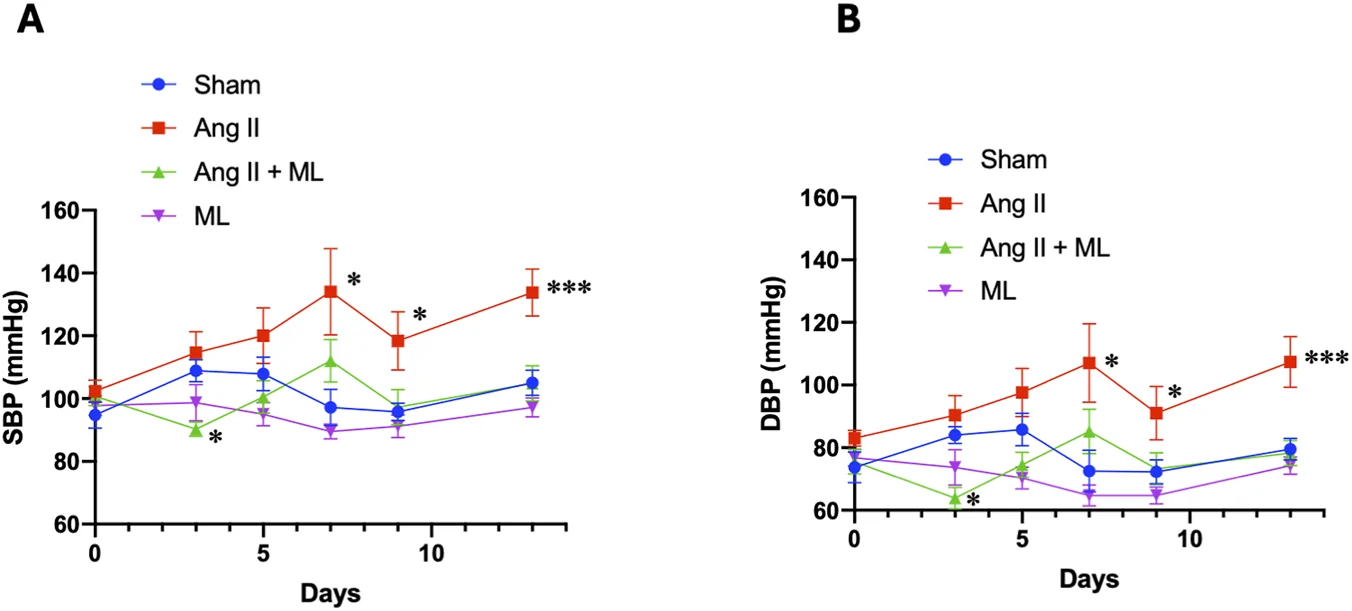
Systolic **(A)** and diastolic **(B)** arterial blood pressure in mice infused with angiotensin II (Ang II) with or without montelukast (ML) for 14 days. Ang II augments systolic, diastolic and mean arterial blood pressure, while ML impairs this effect. **p < 0.01, ***p < 0.001 versus sham.

As shown in Figure 1A, Ang II infusion significantly increased systolic blood pressure on day 7, day 9, and day 13 as compared to the sham group. In contrast, mice receiving Ang II plus ML maintained systolic pressure at values like control animals. As shown in Figure 1B, comparable effects were also observed for diastolic blood pressure. Similarly, Montelukast prevented the increases in diastolic blood pressure in Ang II infused mice.

#### 3.1.2 Body weight, urinary volume and 24-h Na^+^, Cl^−^ and K^+^ excretion in Ang II and Ang II plus montelukast treated mice

**FIGURE 2:**
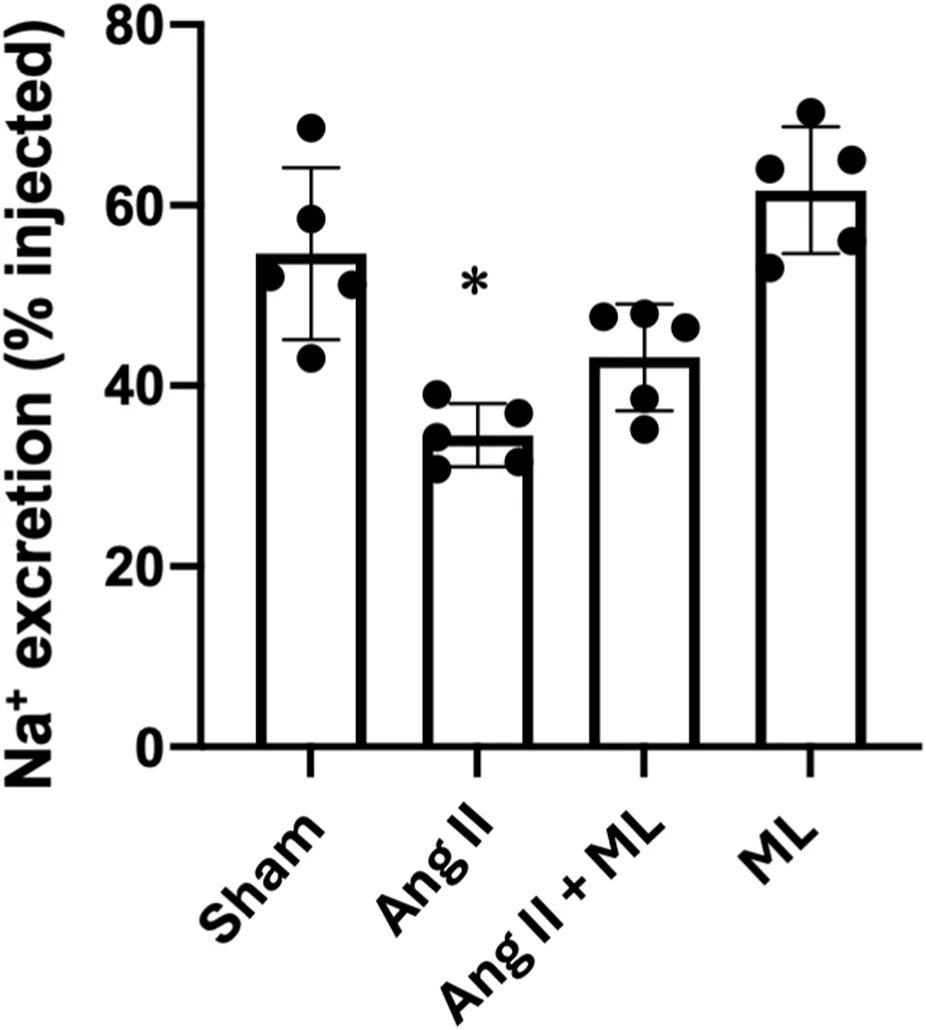
Natriuretic responses to a saline load. Na^+^ excretion (% of injected Na^+^) was significantly reduced in mice infused with Ang II compared with sham controls. Pharmacological blockade of OXGR1 with montelukast (Ang II + ML) attenuated the antinatriuretic effect of Ang II, restoring Na^+^ excretion toward control values, while ML alone did not alter Na^+^ excretion. Data are presented as mean ± SEM with individual values shown; *P < 0.05 versus sham.

The effect of ML on body weight, urinary volume and electrolyte excretion in controls and Ang II-infused mice was determined at the end of the protocol. We did not find any changes in all the parameters evaluated (Table 1). No changes were observed in food, water intake and urinary PH (not shown). One day before the end of the treatment (day 13), mice were injected with saline 10% body volume and excreted Na^+^ was analyzed as the percentage of sodium load that was excreted at 6 h. We found a significant difference between Ang II and sham mice but not when compared Ang II plus ML (Figure 2) This data demonstrate that natriuretic responses were more rapid in Ang II infused mice treated with ML.

**TABLE 1:** Body weight, urinary electrolytes, at 13 days. Body weight and urinary electrolyte excretion were not significantly affected by Ang II, montelukast (ML), or their combination. n = 4–6.

​	Sham	Ang II	Ang II + ML	ML
Body weight (g)	26.45 ± 1.34	25.91 ± 1.27	24.70 ± 1.97	25.38 ± 1.75
Na^+^ (mEq/24h)	142.38 ± 26.52	148.26 ± 26.18	139 ± 22.57	150.91 ± 29.12
K^+^ (mEq/24h)	407.92 ± 72.45	354.72 ± 83.26	350.94 ± 85.97	366.37 ± 55.65
Cl^−^ (mEq/24h)	290.12 ± 57.34	262.91 ± 51.28	245.50 ± 28.72	246.30 ± 33.93

#### 3.1.3 OXGR1 antagonism prevented the increased protein expression of pendrin αENaC and the (pro)renin receptor in Ang II infused mice

**FIGURE 3:**
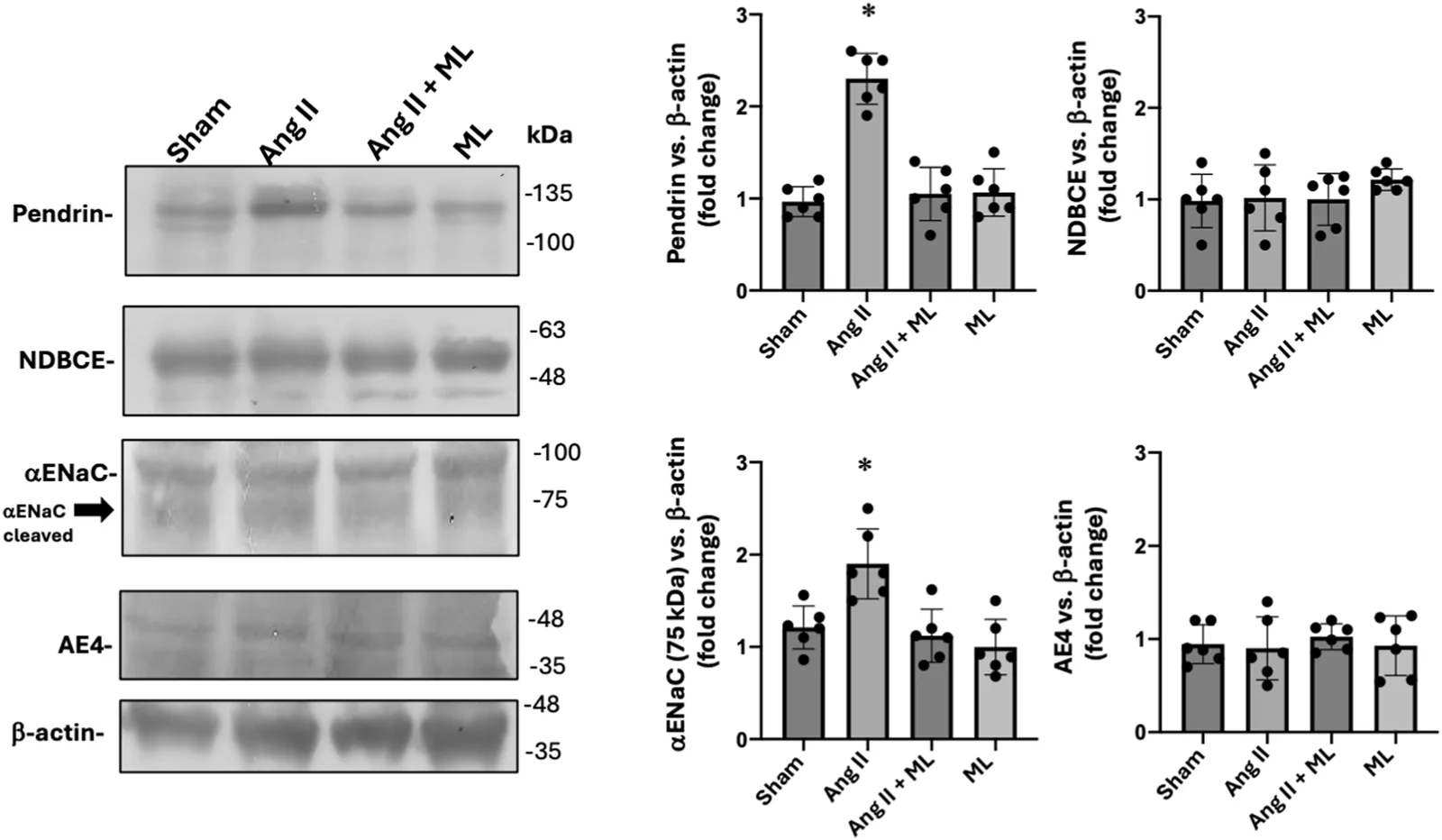
Effect of Ang II infusion and OXGR1 inhibition on pendrin, NDBCE and αENaC protein abundances. Representative immunoblots (left panels) and densitometric analyses (right panels) showing renal protein abundance of key intrarenal on pendrin, NDBCE and the cleaved active form of αENaC in the experimental groups. Chronic Ang II infusion increased the abundance of pendrin and αENaC compared with sham-treated controls. Pharmacological inhibition of OXGR1 with montelukast (ML) attenuated these responses, preventing the Ang II-induced increases in protein expression. Quantitative data are presented as mean ± SEM, normalized to the corresponding loading control β-actin and expressed relative to the control group. Individual data points are shown for each animal. *p < 0.05, n = 6.

Differences in blood pressure and Na^+^ handling observed in Ang II infused mice treated or not with OXGR1 antagonist prompted us to examine Na^+^ related transporters pendrin and NDCBE. Due to recent evidence demonstrating that OXGR1 can enhance intratubular RAS activity and αENaC abundance, we also examined its protein levels by immunoblot. As shown in Figure 3, Pendrin abundance was augmented in Ang II-infused mice as compared to sham. No differences were found by comparing sham and Ang II + ML. The cleaved (75 kDa) form of αENaC (Gonzalez-Villalobos et al., 2013; Prieto et al., 2017) was augmented by Ang II-infusion, however ML treatment blunted this effect. No differences were observed in the abundance of the NDCBE in all treatments.

**FIGURE 4:**
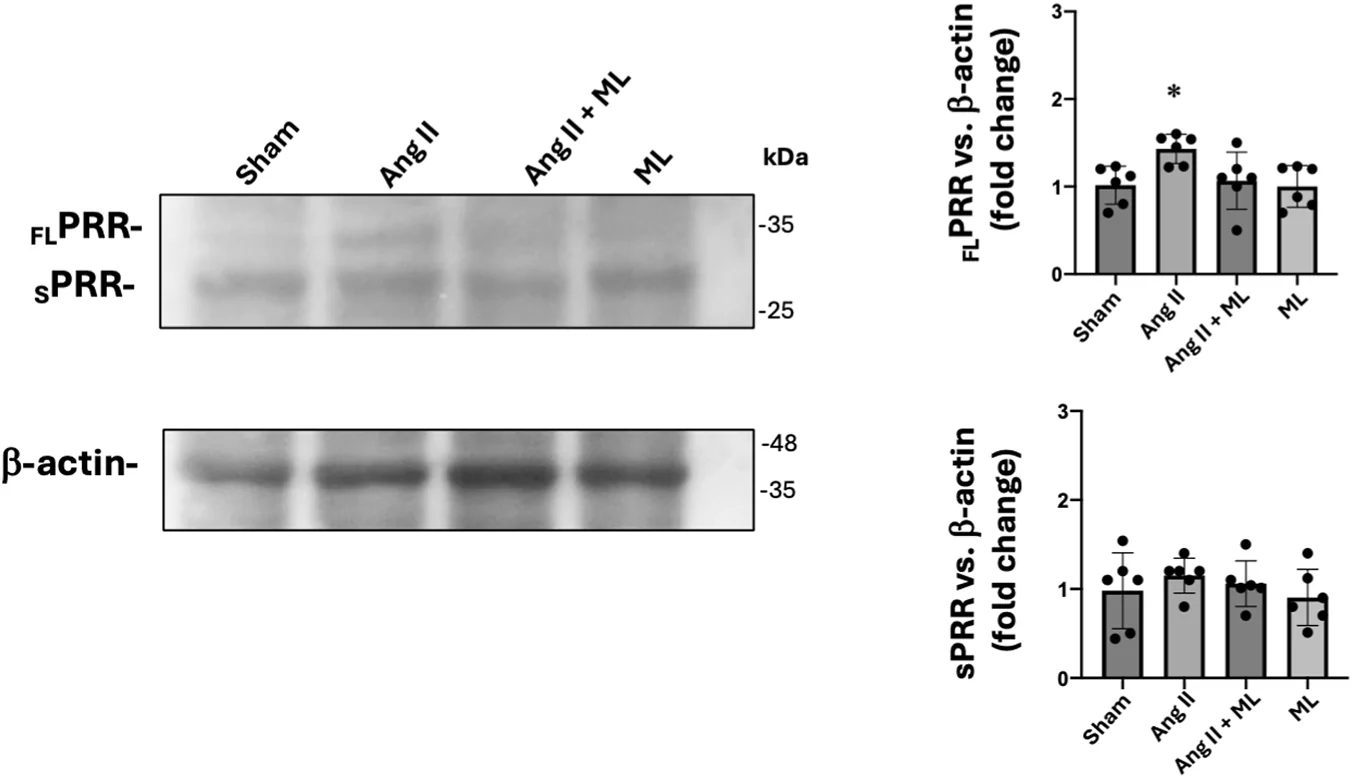
Effects of Ang II infusion and OXGR1 blockade on full-length (membrane-bound) and soluble form of the PRR protein abundance in the kidney. Representative immunoblots (left) and densitometric analyses (right) of full-length (FL)-PRR and soluble PRR (sPRR) protein abundance in control mice, Ang II-infused mice, and Ang II-infused mice treated with montelukast (ML). Ang II infusion significantly increased FL-PRR abundance compared with controls. In contrast, ML treatment prevented the Ang II-induced increase in FL-PRR, maintaining protein levels near control values. No significant differences were observed in sPRR abundance among experimental groups. *p < 0.05, n = 6.

To investigate whether PRR is regulated by OXGR1 signaling, we examined the effects of montelukast (ML) on both forms of the (pro)renin receptor (PRR): the full-length PRR (FL-PRR), a 35–39 kDa single transmembrane receptor that binds both renin and prorenin and initiates intracellular signaling pathways, and the soluble PRR (sPRR), a ∼28 kDa fragment generated by proteolytic cleavage of the N-terminal extracellular domain and released into the extracellular compartment (Nguyen et al., 2002). As shown in Figure 4, chronic Ang II infusion significantly increased FL-PRR abundance, whereas sPRR levels remained unchanged. Notably, ML treatment prevented the Ang II-induced increase in FL-PRR expression without affecting sPRR abundance. These findings suggest that OXGR1 signaling selectively regulates membrane-associated PRR expression during Ang II-dependent hypertension.

#### 3.1.4 Histochemical analysis of pendrin and αENaC distribution in kidney tissues of control mice, Ang II-infused and Ang II plus montelukast treatment

**FIGURE 5:**
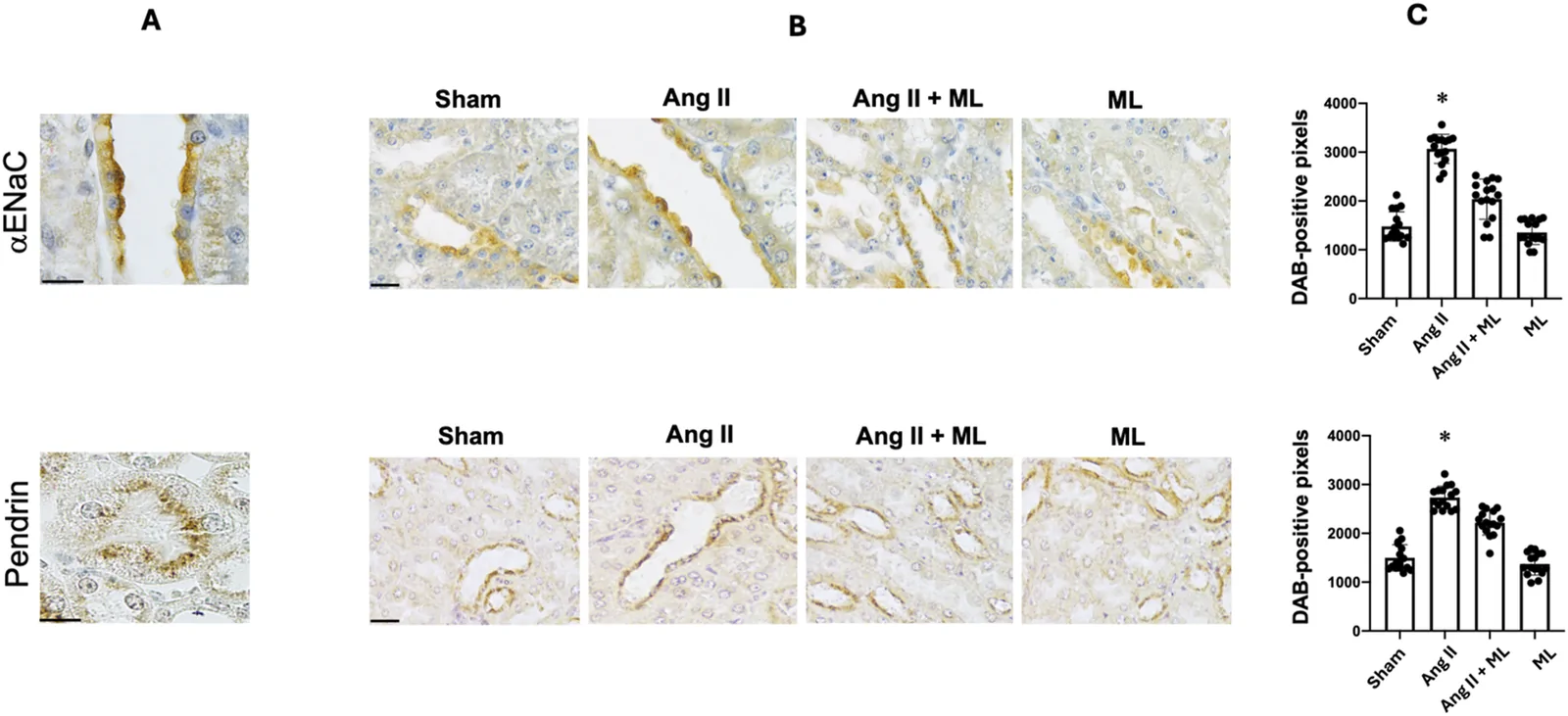
Immunohistochemical localization of pendrin and αENaC in renal collecting ducts. Representative immunohistochemical images showing pendrin and αENaC distribution in kidney sections from the indicated experimental groups. **(A)** High-magnification images (100X) illustrating prominent apical membrane staining of pendrin and αENaC in collecting duct epithelial cells from control mice. **(B)** Positive immunoreactivity is visualized as a brown diaminobenzidine (DAB) precipitate, whereas nuclei are counterstained with hematoxylin. In control kidneys, staining was localized in the cytoplasm and apical, however in Ang II-infused mice the DAB-positive pixels were augmented along with more prominent apical membrane intensity. **(C)** DAB-positive pixels were less intense in Ang II plus ML treatment. Images are representative of 4-6 fields per animal of each group. *p < 0.05 vs. sham. Scale bar 20 μm.

Since Ang II upregulates pendrin expression in intercalated cells of the collecting duct (Gonzalez-Villalobos et al., 2013) and αENaC (Beutler et al., 2003) we investigated whether OXGR1 may have a role in the regulation of pendrin and αENaC under our experimental conditions. Both proteins showed prominent apical membrane staining in collecting duct epithelial cells (Figure 5A). Chronic Ang II infusion causes a focal upregulation of αENaC and pendrin intensity in the collecting ducts (Figure 5B). A significant difference was found between Ang II-infused mice and sham groups; however, no statistical differences were found in Ang II + ML or ML groups when compared to control mice (sham) (Figure 5C).

#### 3.1.5 OXGR1 antagonism prevented Ang I- induced intrarenal Ang I and Ang II increase

We first assessed intrarenal Ang I levels as an index of tissue renin activity following 14 days of Ang II infusion in the presence or absence of the OXGR1 antagonist montelukast (ML). Intrarenal Ang I levels (fmol·mg protein^−1^) were significantly increased in both Ang II-infused mice (1.5 ± 0.1 vs. 1.1 ± 0.1 in sham, p < 0.01) and Ang II + ML-treated mice (1.4 ± 0.1 vs. 1.1 ± 0.1 in sham, p < 0.01). No significant differences were observed between the sham and ML groups. Similarly, intrarenal Ang II levels (fmol·mg protein^−1^) were significantly elevated in Ang II-infused mice compared with sham controls (1.5 ± 0.3 vs. 0.8 ± 0.1, p < 0.05). In contrast, Ang II levels in the Ang II + ML group were not significantly different from those in sham mice, suggesting that OXGR1 blockade prevents intrarenal Ang II accumulation despite persistent Ang I generation. No significant differences were detected in intrarenal Ang 1-5 levels among the experimental groups; however, Ang 1-7 was significantly different from control mice in the Ang II + ML group (1.3 ± 0.1 vs. 1.0 ± 0.1 in sham, p < 0.05). Interestingly, renal ACE activity (nmol·min^−1^·mg protein^−1^) was markedly increased in both Ang II-infused mice (11.5 ± 7.1 vs. 0.5 ± 0.1 in sham, p < 0.01) and Ang II + ML-treated mice (22.2 ± 12.1 vs. 0.5 ± 0.1 in sham, p < 0.01). These findings indicate that OXGR1 blockade dissociates the increase in ACE activity from intrarenal Ang II accumulation, suggesting that OXGR1 signaling is required for the maintenance or amplification of intrarenal Ang II levels during chronic Ang II infusion.

### 3.2 Protocol 2: chronic Ang II infusion in OXGR1 knockout mice

#### 3.2.1 The increase in blood pressure induced by chronic Ang II infusion is blunted in OXGR1^−/−^ mice

**FIGURE 6:**
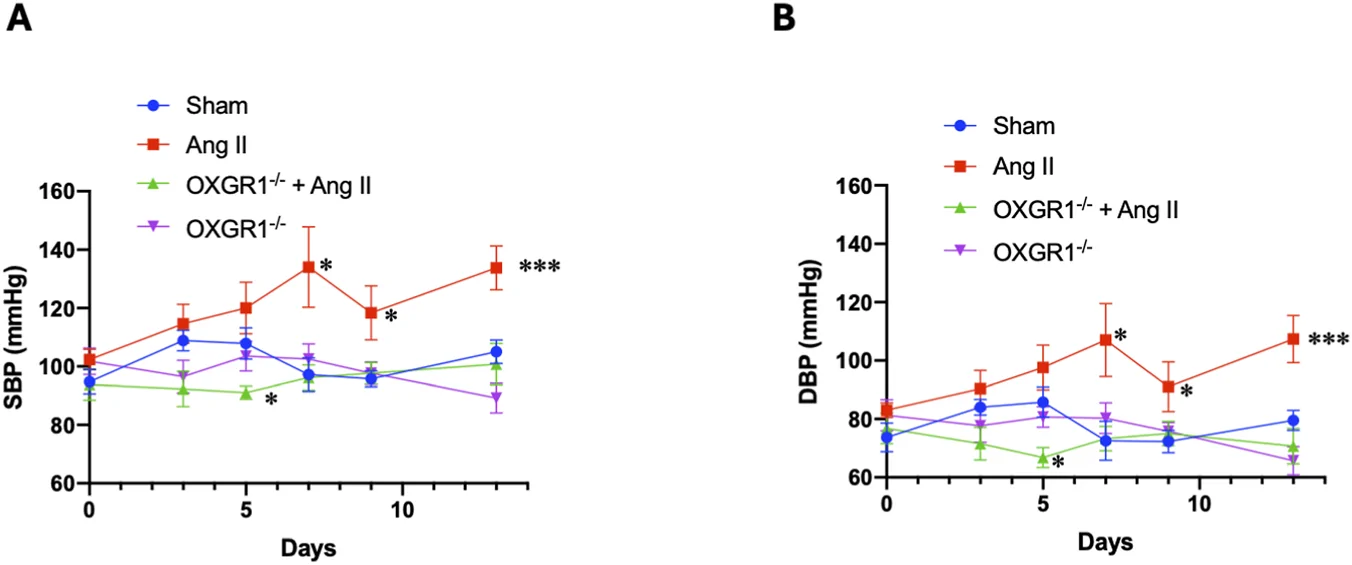
Systolic **(A)** and diastolic **(B)** blood pressure overview in wild type or OXGR1^−/−^ with or without Ang II infusion. Systolic and diastolic blood pressure was augmented in Ang II infused wild type mice but not in OXGR1^−/−^ mice t days 7, 9 and 13. *p < 0.05; **p < 0.01; ***p < 0.001 versus sham.

Starting at 7 days, Ang II infusion caused a significant increase in systolic and diastolic blood pressure of WT mice (Figure 6). In contrast, OXGR1^−/−^ mice did not show significant differences in systolic (Figure 6A) or diastolic (Figure 6B) blood pressure after Ang II infusion. Interestingly, at day 5 Ang II infusion caused a significant reduction in systolic and diastolic blood pressure in OXGR1^−/−^ mice as compared to the sham group (Systolic: 91 ± 5 mmHg vs. 106 ± 4 mmHg, p < 0.05; Diastolic: 66 ± 4 mmHg vs. 85 ± 5 mmHg, p < 0.05). No differences were observed between the OXGR1^−/−^ group and wild type sham group.

#### 3.2.2 Body weight, urinary volume and 24-h Na^+^, Cl^−^ and K^+^ excretion in wild type and OXGR1^−/−^ mice infused with Ang II

**FIGURE 7:**
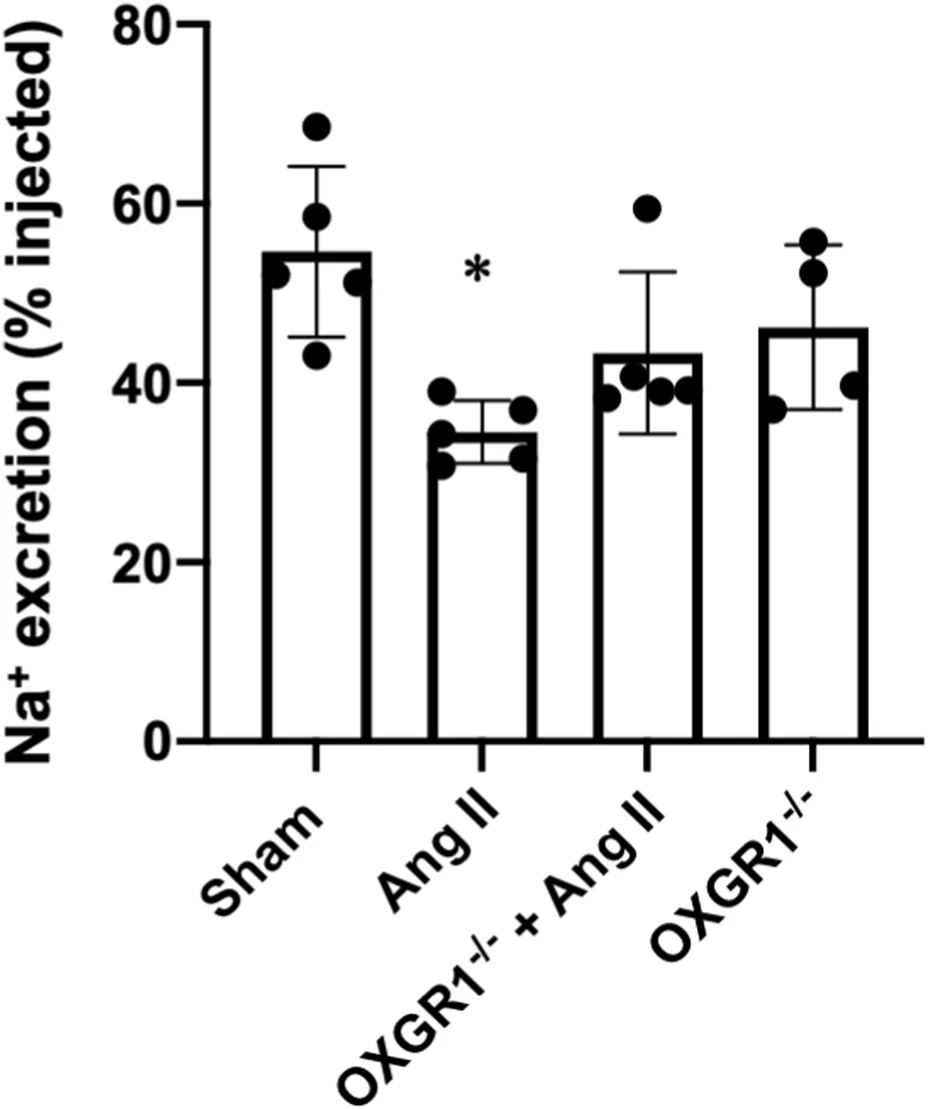
Natriuretic responses to a saline load in wild type and OXGR1^−/−^ mice after 6 h. Chronic Ang II infusion significantly reduced Na^+^ excretion compared with sham mice, indicating an antinatriuretic response. Genetic deletion of OXGR1 prevented the Ang II-induced reduction in Na^+^ excretion, restoring values toward those observed in sham controls, whereas OXGR1^−/−^ mice alone showed no differences from sham. Data are presented as mean ± SEM with individual values shown; *P < 0.05 versus sham.

No differences were found on the global Na^+^, Cl^−^ or K^+^ excretion at day 14 using 24-h metabolic cage in sham wild type, Ang II or OXGR1^−/−^ mice infused with Ang II (Table 2). Next, we evaluated the response to an acute saline challenge in Ang II-infused OXGR1^−/−^ mice on day 13. While Ang II significantly blunted the natriuretic response in wild-type mice, this effect was abolished in OXGR1^−/−^ mice, which maintained normal sodium excretion following saline loading (Figure 7). No significant differences were observed among the experimental groups at the preceding urine collection time points.

**TABLE 2:** Body weight and urinary electrolyte excretion at the end of the treatment. Body weight and urinary electrolyte excretion were not significantly altered by Ang II infusion in OXGR1^−/−^ mice. n = 4–6.

​	Sham	Ang II	KO Ang II	OXGR KO
Body weight (g)	26.45 ± 1.34	25.91 ± 1.27	26.42 ± 1.95	24.68 ± 3.81
Na^+^ (mEq/24h)	142.38 ± 26.52	148.26 ± 26.18	167.31 ± 39.57	186.91 ± 33.12
K^+^ (mEq/24h)	407.92 ± 72.45	354.72 ± 83.26	301.15 ± 64.81	326.16 ± 9.52
Cl^−^ (mEq/24h)	290.12 ± 57.34	262.91 ± 51.28	255.61 ± 82.26	352.70 ± 67.86

#### 3.2.3 Ang II mediated increases in protein abundances of αENaC, pendrin is blunted in OXGR1^−/−^ mice

**FIGURE 8:**
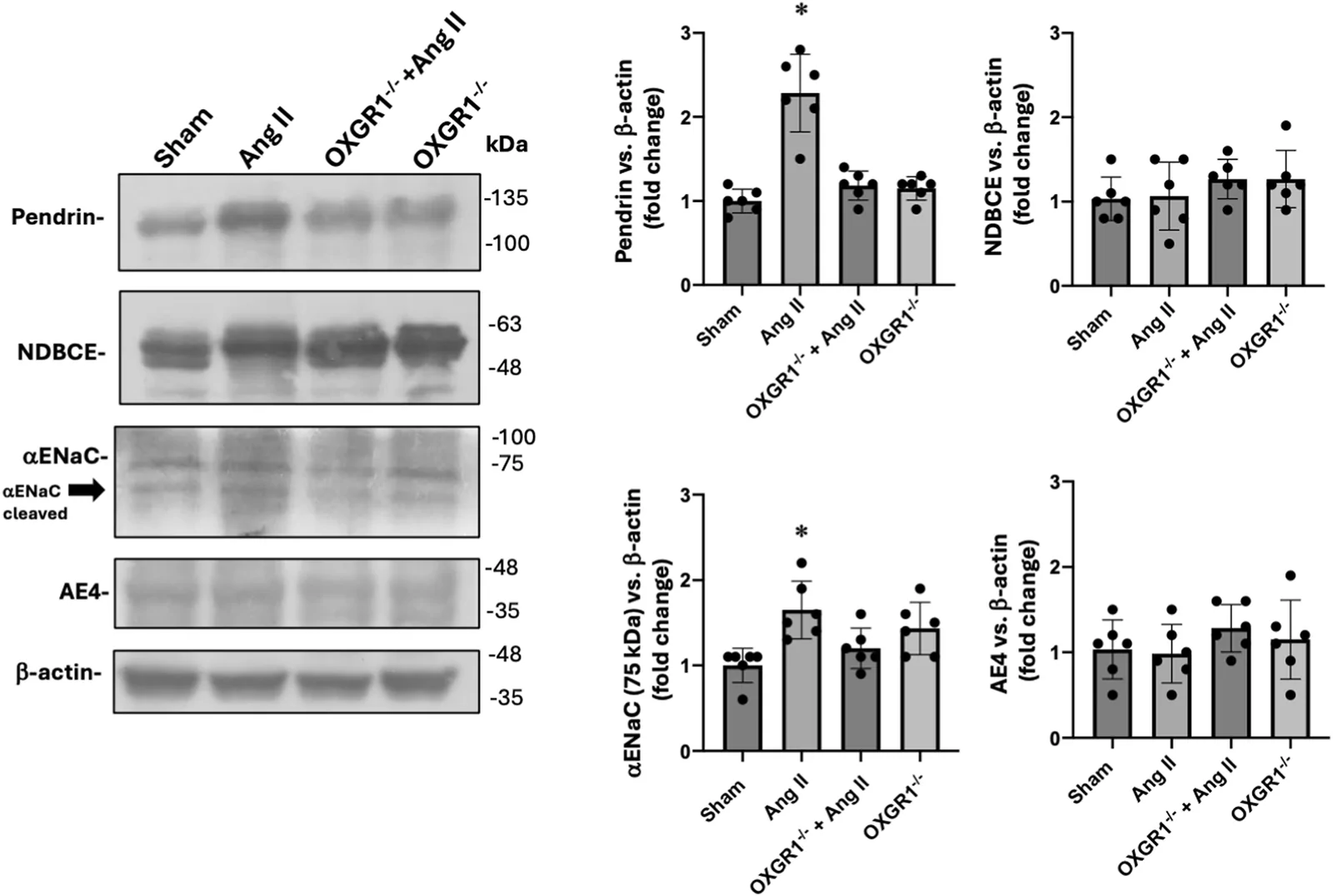
Effect of Ang II infusion and OXGR1 deletion on pendrin, NDBCE and αENaC protein abundances. Representative immunoblots (left) and densitometric analyses (right) showing renal protein abundance of pendrin, NDBCE, and the cleaved active form of αENaC in wild-type Sham, wild type Ang II-infused, OXGR1^−/−^ + Ang II-infusion and OXGR1^−/−^ mice. Compared with sham animals, Ang II infusion increased the abundance of pendrin and the cleaved αENaC, as indicated by the arrow in the left panel. In contrast, Ang II infusion did not modify pendrin abundance in OXGR1-deficient mice. Data are presented as mean ± SEM, with individual values shown for each animal. *p < 0,05 versus sham, n = 6.

As shown in Figure 8, we found no difference in pendrin abundance between sham and OXGR1^−/−^ mice. Ang II infusion caused a 2-fold increase in renal pendrin protein abundance (Ang II: 2.3 ± 0.4 vs. sham: 1.0 ± 0.1-fold change, p < 0.05). In contrast, OXGR1^−/−^ mice were protected from Ang II-induced pendrin upregulation (OXGR1^−/−^ Ang II: 1.1 ± 0.2 vs. sham: 1.0 ± 0.1 p = non-significant). Similarly, the 75 kDa band of αENaC was augmented in Ang II infused mice (Ang II: 1.7 ± 0.1 vs. sham: 1.0 ± 0.1, fold change, p < 0.05); the increase in αΕNAC abundance after Ang II infusion was prevented in OXGR1^−/−^ mice (OXGR1^−/−^ Ang II: 1.2 ± 0.2 vs. sham: 1.0 ± 0.1-fold change, p = non-significant). Contrasting with Pendrin and αENaC, NDCBE and AE4 protein abundance were not altered by Ang II infusion or by the genetic ablation of OXGR1.

#### 3.2.4 Ang II mediated increases in protein abundances of the full-length of the prorenin receptor was blunted in OXGR1^−/−^ mice

**FIGURE 9:**
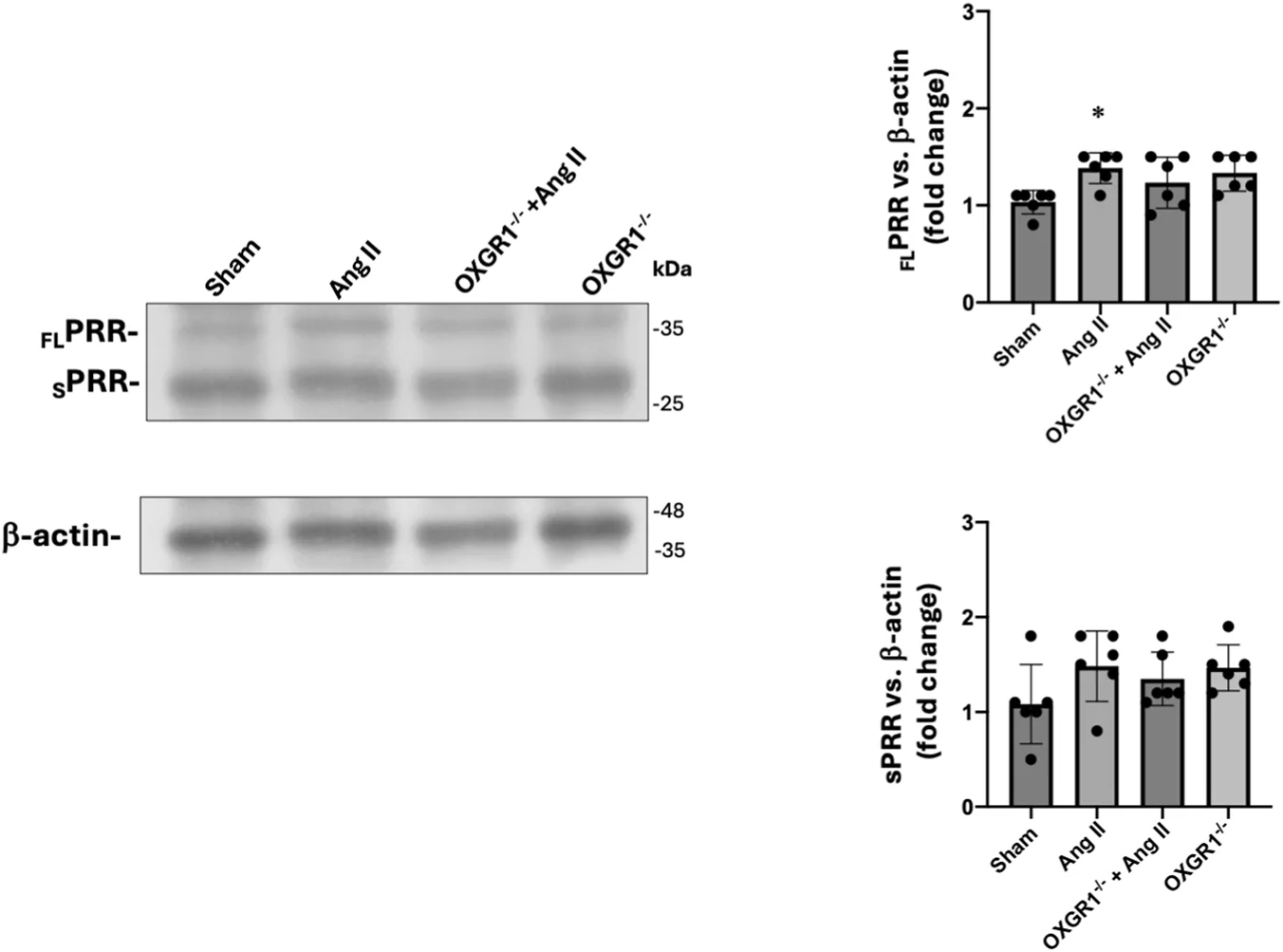
Representative immunoblots (left) and densitometric analyses (right) of the membrane-bound full-length (FL)-prorenin receptor (PRR) and soluble PRR (sPRR) protein abundance in renal tissues from WT and OXGR1^−/−^ mice following 14 days of Ang II infusion. Ang II significantly increased FL-PRR abundance in WT mice, whereas this response was absent in OXGR1^−/−^ mice. In contrast, sPRR levels remained unchanged across all experimental groups. Data are presented as mean ± SEM, and representative immunoblots are shown adjacent to their corresponding quantification panels. *p < 0.05 versus sham, n = 6.

Since membrane-bound PRR (full-length, _FL_PRR 37 kDa) can bind renin and prorenin enhancing their catalytic activities, we evaluated changes in PRR protein abundance. As shown in Figure 9, Ang II mice showed a significant increase in the _FL_PRR form (Ang II: 1.4 ± 0.1 vs. sham: 1.0 ± 0.1-fold change, p < 0.05). In contrast, _FL_PRR abundance was not affected by Ang II infusion in OXGR1^−/−^ mice. No changes were observed in the 28 kDa, which is the soluble form (sPRR).

#### 3.2.5 Ang II mediated increase in αENaC and pendrin immunostaining are blunted in OXGR1 knockout mice

**FIGURE 10:**
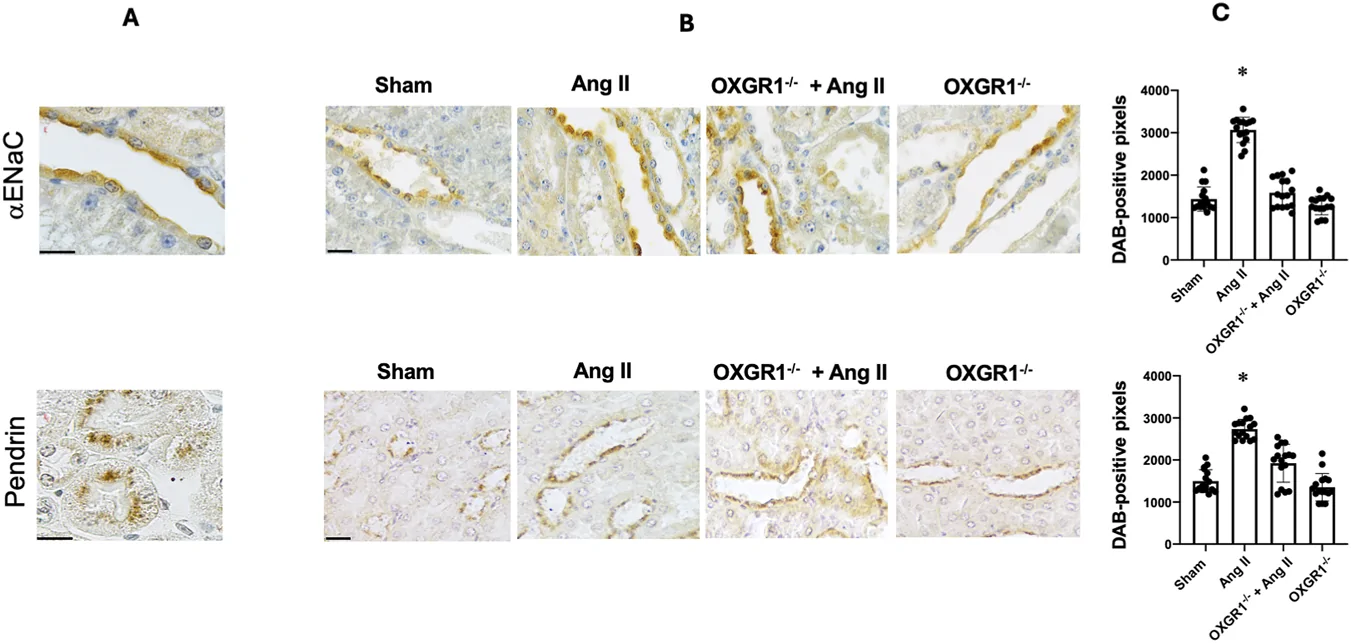
Representative immunohistochemical staining of αENaC and pendrin and in renal collecting ducts from WT and OXGR1^−/−^ mice after 14 days of Ang II infusion. **(A)** High-magnification images reveal distinct cell-specific localization of αENaC cytoplasmic and apical aspects in principal cells (PC), while pendrin was mainly localized in the apical side of intercalated cells (IC). **(B)** Pendrin and αENaC immunoreactivity were augmented in the collecting ducts of wild-type mice infused with Ang II. OXGR1^−/−^ infused with Ang II showed less intensity of DAB-positive pixels. **(C)** The right panels show quantification of DAB-positive pixels. Images are representative of 4-6 fields per animal of each group. *p < 0.05 vs. sham. Scale bar 20 μm.

We evaluated the effects of Ang II infusion and OXGR1 deficiency on renal tissue ENaC and pendrin. As shown in Figure 10A, both αENaC and pendrin were detected in the cytoplasmic and apical regions of collecting duct cells in WT mice. In WT mice, Ang II infusion increased the αENaC immunostaining area. In contrast, in OXGR1^−/−^ mice did not modify αENaC immunostaining area (Figures 10B,C). Similarly, Ang II infusion enhanced pendrin immunoreactivity in WT mice, whereas OXGR1 deficiency prevented the increase in pendrin (Figures 10B,C). Also, the apical localization of both αENaC and pendrin was less evident in OXGR1^−/−^ mice compared with WT animals, irrespective of Ang II infusion.

#### 3.2.6 Intrarenal Ang I and Ang II are increased in wild-type mice infused with Ang II but not in Ang II-OXGR1^−/−^ infused

To determine whether OXGR1 genetic ablation affects Ang II-dependent increase of intrarenal Ang I and Ang II levels, we measured intrarenal angiotensin peptide levels in OXGR1^−/−^ mice following 14 days of Ang II infusion. Intrarenal Ang I levels (fmolmg protein^−1^) were significantly elevated in Ang II-infused OXGR1^−/−^ mice compared with sham controls (1.4 ± 0.1 vs. 1.1 ± 0.1, p < 0.01). No significant differences were observed between WT and OXGR1^−/−^ control mice. In contrast, intrarenal Ang II levels in Ang II-infused OXGR1^−/−^ mice were not significantly different from those in sham animals (1.1 ± 0.2 vs. 0.8 ± 0.1), despite the increase in Ang I. These findings suggest that OXGR1 is required for intrarenal Ang II accumulation during chronic Ang II infusion. Consistent with the results obtained in Protocol 1, no significant differences were detected in Ang-1-7 or Ang-1-5 levels among the experimental groups.

## 4 Discussion

Our findings provide pharmacological and genetic evidence supporting that OXGR1 is necessary for the hypertensive response to Ang II-infusion. Both the pharmacological blockade with montelukast and the genetic deletion of OXGR1 precented the increase in blood pressure in response to Ang II infusion. Importantly, the disruption of OXGR1 signaling prevented the antinatriuretic effects of Ang II and the upregulation of pendrin and αENaC, two key transport proteins involved in distal nephron NaCl reabsorption. These observations demonstrate that OXGR1 is a necessary component for the renal actions of Ang II, linking metabolic sensing with intrarenal RAS activation and sodium-retaining mechanisms.

Our data also suggest that renal metabolic intermediates may be responsible for active modulation of epithelial transport processes, beyond acid-base regulation in the distal nephron (Tokonami et al., 2013). The α-ketoglutarate, traditionally viewed as a tricarboxylic acid cycle metabolite, appears to operate as a luminal signaling molecule able to activate OXGR1 along the distal nephron (He et al., 2004; Wittenberger et al., 2002). Under conditions associated with altered systemic or cellular metabolism, increased availability of α-ketoglutarate within the tubular lumen may provide a mechanism by which metabolic modulation in the proximal tubule influence distal transport function (Efimov et al., 1983; Guan et al., 2013). The physiological role of this pathway becomes relevant when considered in the context of intrarenal RAS activation. It is well established that Ang II-dependent hypertension is sustained, in part, by increased intratubular Ang II formation, which enhances Na^+^ reabsorption through multiple mechanisms (Prieto et al., 2021; Gonzalez et al., 2024; Lara et al., 2022). The collecting duct serves as a key effector site where local angiotensin generation and receptor signaling converge to regulate epithelial Na^+^ channel (ENaC) activity (Prieto et al., 2021; Mironova et al., 2012; Ramkumar et al., 2014). Here, we showed that αENaC is augmented after 14 days of Ang II infusion, along with increased activity of ACE. In contrast to our previous studies in using a model of renovascular hypertension (2K1C), we did not observe an attenuation of ACE activity in mice simultaneously infused with Ang II and montelukast. Furthermore, it seems that in this experimental model, ACE activity is further augmented during OXGR1 antagonism. These findings suggest that OXGR1 regulates αENaC via intratubular RAS pathways distinct from ACE-dependent processes and independent of Ang II amplification.

Traditionally, Na^+^ reabsorption in the collecting duct has been attributed primarily to principal cells through the ENaC. Intercalated cells were historically associated with acid-base regulation, however, recent studies have demonstrated that they also contribute to NaCl transport through functional interaction with principal cell transport mechanisms (Wall et al., 2020). Pendrin is a Cl^−^/HCO_3_^−^ exchanger expressed in type B and non-A/non-B intercalated cells of the collecting duct; it participates in electroneutral NaCl reabsorption through cooperation with the Na^+^-driven chloride/bicarbonate exchanger NDCBE. This transport mechanism allows intercalated cells to contribute significantly to distal Na^+^ balance (Verlander et al., 2006). Indeed, it has been shown that pendrin activity influences Na^+^ reabsorption in neighboring principal cells by modulating ENaC abundance and function, thereby integrating chloride and Na^+^ transport processes within the collecting duct (Wall et al., 2020; Kim et al., 2007). Then, pendrin-mediated Cl^−^ transport has emerged as a critical regulator of distal NaCl absorption (Verlander et al., 2006). At the cellular level, the coordinated regulation of transporters in principal and intercalated cells emerges as a critical determinant of Na^+^ balance. While ENaC in principal cells mediates electrogenic Na^+^ entry, pendrin expressed in intercalated cells supports electroneutral chloride reabsorption (Lazo-Fernandez et al., 2018; Grimm and Welling, 2017). Increasing evidence indicates that these two systems do not operate independently but are functionally coupled (Kim et al., 2007; Pham et al., 2020). The current data showing that disruption of OXGR1 signaling prevents the upregulation of both pendrin and αENaC support the concept that a common upstream pathway coordinates their activation (Kim et al., 2007; Pech et al., 2015; Wall and Weinstein, 2013). Furthermore, changes in luminal bicarbonate concentration associated with pendrin activity may directly influence ENaC expression and function (Kim et al., 2007). Thus, the coordinated regulation of these transporters represents a mechanism to enhance Na^+^ reabsorption, and disruption of OXGR1 signaling might be able to alter this integrated response. We have previously demonstrated that aldosterone levels are significantly increased at day 14 in both rats and mice infused with Ang II, and that mineralocorticoid receptor (MR) antagonism prevents the Ang II-induced upregulation of αENaC. However, MR blockade does not prevent the increase in renin abundance or the stimulation of PRR expression observed during chronic Ang II infusion (Gonzalez et al., 2011). These findings suggest that, although aldosterone contributes to αENaC regulation, activation of the intrarenal RAS occurs independently of MR signaling and epithelial sodium channel activity. Based on these observations, we propose that the OXGR1–PRR–Ang II axis represents a distinct regulatory pathway. Such a mechanism could explain how local Ang II generation and PRR activation remain elevated despite pharmacological inhibition of MR signaling.

Since OXGR1 is a Gq coupled receptor, the blunted upregulation of pendrin and αENaC abundance observed in montelukast treated mice and in OXGR1-deficient mice infused with Ang II, suggests that downstream signaling pathways mediated by Gq-dependent pathways which may be responsible for the coordinated activation of distal nephron Na^+^ transport. One plausible mechanism is that OXGR1 regulates PRR in intercalated cells. In experimental type 1 diabetes, we have reported that OXGR1 regulates PRR expression *in vivo* and in cultured collecting duct cells (Guerrero et al., 2021; Cardenas et al., 2024). OXGR1 signals through protein kinase C (PKC) and Ca^2+^ (Wittenberger et al., 2002), acting on intercalated cell metabolism. Interestingly, we have shown that PRR can be regulated by Ca^2+^ and PKC in collecting duct cells under Ang II infusion and high salt diet (Gonzalez et al., 2014), suggesting that OXGR1-PKC-Ca^2+^ may mediate the regulation of PRR expression.

Activation of membrane bound (full length) PRR enhances the catalytic activity of tubular prorenin and renin, thereby promoting local Ang I generation (Nguyen, 2006). In the presence ACE along the nephron, increased Ang I can be efficiently converted to Ang II, leading to enhanced ENaC activity. Furthermore, the absence of PRR causes Na^+^ loss, urine concentration defect and reduced protein abundance of αENaC in collecting ducts (Prieto et al., 2017; Ram et al., 2015). Then, coordinated actions of OXGR1-mediated PRR, intrarenal RAS and pendrin abundance and function would ultimately favor increased Na^+^ reabsorption in the distal nephron contributing to the regulation of extracellular volume and blood pressure. Because ENaC and pendrin are expressed in different cell types and thus cannot interact directly, it is likely that luminal pH may also contribute to ENaC regulation. Indeed, global pendrin gene ablation does not modify ENaC abundance in non-renal tissues (Kim et al., 2007). It has been reported that changes in pH in the collecting duct may alter ENaC activity. Increases in apical HCO_3_^−^ concentration augmented ENaC abundance and function on collecting duct cell monolayers. Thus, it is suggested that pendrin modulates ENaC at least in part by raising luminal HCO_3_^−^ concentration (Kim et al., 2007). Notably, Cl^−^ excretion was not altered in either montelukast-treated or OXGR1^−/−^ mice infused with Ang II, despite the well-established role of pendrin as a Cl^−^/HCO_3_^−^ exchanger that mediates electroneutral NaCl reabsorption in coordination with the Na^+^-driven Cl^−^/HCO_3_^−^ exchanger NDCBE. These findings suggest that maintenance of Cl^−^ balance in these experimental groups may involve compensatory mechanisms operating in other nephron segments, thereby preserving overall Cl^−^ handling despite the disruption of OXGR1-dependent regulation of the pendrin/NDCBE pathway.

Importantly, the absence of hypertensive responses in mice with chronic pharmacological blockade of OXGR1 and the genetic ablation of OXGR1 suggests that this receptor may also modulate systemic or intrarenal RAS activity acting downstream of Ang II. Another possibility is that OXGR1 may participate in a feed-forward mechanism that may modulate and reinforce Na^+^-retaining pathways. Gonzalez-Villalobos et al., previously demonstrated that mice lacking kidney ACE showed similar expression of distal Na^+^ channel including pendrin, however, Ang II infusion triggered an abundance of the cleaved forms of αENaC subunit, and increased abundance of pendrin (Gonzalez-Villalobos et al., 2013). This finding agrees with our results and suggests that intrarenal ACE-may not be a limiting step for the regulation of the abundance of distal Na^+^ transporters. Furthermore, the intrarenal increases in Ang I with Ang II infusion suggest that ACE may participate in different enzymatic pathways for the degradation of Ang I and Ang II which may counteract intrarenal RAS promoting natriuretic and vasodilator responses. Increased Ang 1-7 in Ang II + ML may exert actions against the Ang II pathway (Patel et al., 2016), however it may also be related with the dose of ML and side effects on CysLT1 receptors, indeed given the fact that CysLT_1_R are also expressed on smooth muscle cells in airways and blood vessels (Kanaoka et al., 2013) the effect of ML may be also related to peripheral hemodynamic effects. Given the expression of OXGR1 in multiple organ systems, including vascular and epithelial compartments (Kanaoka et al., 2013; Diehl et al., 2016), systemic effects of OXGR1 antagonism may contribute to the observed phenotype. In a previous study, Omede et al., identified OXGR1 as a novel regulator of pathological cardiac hypertrophy via the regulation of the STAT3, then pharmacological inhibitors for this receptor may be very useful in the development of novel therapeutic approach for pathological cardiac hypertrophy (Omede et al., 2016). These findings suggest that OXGR1 may also have an impact on the cardiovascular system, beyond the renal role on fluid and electrolyte balance. Although we have previously demonstrated that direct exposure of cultured collecting duct cells to α-ketoglutarate increases intracellular Ca^2+^ signaling and PRR expression (Guerrero et al., 2021), direct *in vivo* assessment of tubular α-ketoglutarate concentrations under physiological and pathological conditions would further strengthen the proposed mechanistic framework. Such measurements would provide valuable insight into the extent to which local α-ketoglutarate availability contributes to OXGR1 activation and subsequent regulation of PRR and intrarenal RAS activity.

One limitation of the present study is blood pressure measurements using the tail-cuff method only. Although this methodology is widely used and validated for longitudinal studies in conscious mice, it has inherent limitations compared with radiotelemetry. Tail-cuff measurements may be influenced by factors such as animal handling, restraint, and stress responses, potentially increasing variability and reducing temporal resolution. In contrast, telemetry allows continuous blood pressure monitoring under freely moving conditions and is considered the gold standard for hemodynamic assessment in rodents. Nevertheless, in the present study extensive acclimation procedures were implemented before data collection to minimize stress-related artifacts, and all experimental groups were subjected to identical measurement conditions. Finally, although the observed effects on major transport pathways are robust, more subtle alterations in other components of the intrarenal RAS may not have been fully captured. Furthermore, it is well established that the RAS is influenced by biological sex and sex hormones (Veiras et al., 2017; Xiong et al., 2026). Estrogen generally shifts the balance of the RAS toward the ACE2/Ang-1–7/Mas receptor and AT2 receptor axes, thereby promoting vasodilation and cardiovascular protection. In contrast, testosterone favors activation of the ACE/Ang II/AT1 receptor pathway, which is associated with sodium retention, vasoconstriction, and adverse cardiovascular outcomes (Medina et al., 2020). Because the present study was conducted exclusively in male mice, the potential influence of sex and hormonal status on OXGR1 signaling, intrarenal RAS activation, and blood pressure regulation remains to be determined. Future studies incorporating both sexes will be necessary to establish whether sex-dependent differences modulate the α-ketoglutarate/OXGR1/PRR signaling axis and its contribution to hypertension.

The present data positions OXGR1 as a potential amplifier of hypertensive signaling within the kidney. From a broader perspective, these findings have important implications for understanding the relationship between metabolic disturbances and hypertension. Conditions such as obesity and diabetes are characterized by alterations in renal metabolism and increased activation of RAS (He et al., 2004; Efimov et al., 1983; Guan et al., 2013; Peti-Peterdi, 2010). The α-ketoglutarate-OXGR1 signaling axis may provide a mechanistic link between these processes, allowing metabolic changes to directly influence Na^+^ handling and blood pressure regulation. In conclusion, the present study identifies OXGR1 as a critical mediator of Ang II–dependent increases in distal nephron Na^+^ transport. By linking metabolic signaling to intrarenal RAS activation and coordinated regulation of pendrin and ENaC, OXGR1 emerges as a key integrator of pathways controlling extracellular volume and blood pressure. These findings expand the current understanding of how metabolic factors influence renal function and suggest that targeting OXGR1 may represent a novel strategy for the treatment of hypertension, particularly in settings associated with metabolic dysfunction.

## Data Availability

The raw data supporting the conclusions of this article will be made available by the authors, without undue reservation.
